# Embryonic Stem Cell Conditioned Medium Supports In Vitro Maturation of Mouse Oocytes

**Published:** 2017

**Authors:** Saber Miraki, Aram Mokarizadeh, Omid Banafshi, Vahideh Assadollahi, Mahdad Abdi, Daem Roshani, Fardin Fathi

**Affiliations:** 1. Department of Anatomy, Faculty of Medicine, Kurdistan University of Medical Sciences, Sanandaj, Iran; 2. Department of Immunology and Hematology, Faculty of Medicine, Kurdistan University of Medical Sciences, Sanandaj, Iran; 3. Cellular and Molecular Research Center, Faculty of Medicine, Kurdistan University of Medical Sciences, Sanandaj, Iran; 4. Social Determinants of Health Research Center, Kurdistan University of Medical Sciences, Sanandaj, Iran

**Keywords:** Assisted reproductive technologies, Embryonic stem cells, Mice, Oocytes

## Abstract

**Background:**

This study aimed to investigate the maturation and fertilization rates of immature mouse oocytes using Embryonic Stem Cell Conditioned Medium (ESCM).

**Methods:**

Germinal Vesicle (GV) stage oocytes were observed in 120 NMRI mice, aged 4–6 weeks. GV oocytes with or without cumulus cells were subjected to IVM in either ESCM, Embryonic Stem Cell Growth Medium (ESGM), or α-minimum essential medium (α-MEM). After recording the Metaphase II (MII) oocyte maturation rate, the oocytes were fertilized *in vitro*. The fertilization success rate was recorded after 24 *hr*. The embryos were maintained in potassium Simplex Optimization Medium (KSOM) for 96 *hr* and allowed to grow until the blastocyst stage. After recording developmental competence, they were transferred into the uteri of pseudopregnant mice and their birth rates were recorded.

**Results:**

No significant difference existed between the maturation rates in α-MEM (68.18%) and ESCM (64.67%; p>0.05), whereas this rate was significantly higher for both α-MEM and ESCM compared to ESGM (32.22%; p<0.05). A significant difference in IVF success rate existed for oocytes grown in α-MEM (69.44%), ESCM (61.53%), and ESGM (0%). A significantly higher developmental competence was observed at the blastocyst stage for oocytes grown in α-MEM (51.2%) compared to ESCM (35%; p<0.05). 17 days after embryo transfer into the uteri of pseudopregnant mice, there was a nonsignficant (p>0.05), similar birth rate between α-MEM and ESCM (47 *vs*. 40%).

**Conclusion:**

ESCM is an effective medium for preantral follicle growth, oocyte maturation, and subsequent embryo development.

## Introduction

*In vitro* culture and maturation of Germinal Vesicle (GV) oocytes is an efficient method to generate mature oocytes. This technique, as an adjuvant treatment for infertility, is of paramount importance for assisted reproductive technology ^1,2^. In recent years, numerous attempts have been made to grow and stimulate immature follicles ^1,3–6^ with the intent to eventually treat infertility attributed to polycystic ovary syndrome, premature ovarian failure, and infertility following cancer treatments ^7,8^.

Although the growth of *in vitro* preantral follicles can result in fertilizable oocytes and successful childbirth ^9^, the technique of In Vitro Maturation (IVM) needs to be further enhanced because GV oocytes have a reasonable ability to mature and reach the Metaphase II (MII) stage. However, only 40 to 80% of the oocytes from IVM become fertilized and pass the early embryonic stage. Only 15% of these are implanted and produce viable embryos after transfer ^10–14^. GV oocytes are the main units of ovaries which produce a suitable environment for the growth and maturation of oocytes. Therefore, these oocytes are a great source for folliculogenesis based studies and embryo production. One of the main challenges for growth and development of *in vitro* follicles is the preparation of an appropriate medium that creates *in vitro* conditions which mostly mimic the complex *in vivo* environment ^15–17^. Embryonic stem cells are an *in vitro* copy of embryonic cells at the blastocyst stage, called the inner cell mass ^18,19^. These cells are pluripotent cells similar to the blastomeres that form embryos which have the capability to differentiate into all cell types *in vivo* ^20–22^.

There are reports that discuss the biological secretions and activated proteins by embryonic stem cells which indicate their capacity to provide an environment with mitogenic factors, growth factors, cytokines, and chemokines ^23–26^. These factors prevent the growth of cancer cells and cardiovascular cell apoptosis, play a role in cardiomyocyte division, and lead to angiogenesis ^23–26^. The conditioned medium secreted by embryonic stem cells can regulate the ultimate fate of these cells ^27,28^.

Undifferentiated mouse ES cells grown in ES cell culture medium produce a number of biologically active cytokines such as Epidermal Growth Factor (EGF), Insulin Growth Factor (IGF-1), IGF-2, Stem Cell Factor (SCF), Leukemia Inhibitor Factor (LIF), and Transforming Growth Factor-β (TGF-β) ^25,29^. According to previous reports ^25,29^, meiotic progress, oocyte growth, cumulus cell proliferation, and numerous other processes associated with IVM are stimulated by the above mentioned growth factors and cytokines. In the present study, the purpose was to determine whether IVM of immature oocytes can be improved with the use of mouse Embryonic Stem Cell Conditioned Medium (ESCM). After subsequent In Vitro Fertilization (IVF) and embryo development, an attempt was made to determine if live pups could be successfully produced.

## Materials and Methods

### Mouse embryonic stem cell culture and preparation of conditioned medium

TT2 embryonic stem cells were maintained in Dulbecco’s modified Eagle’s medium (DMEM) with 15% Fetal Bovine Serum (FBS, Gibco), 1 *mM* sodium pyruvate (Gibco), 1% non-essential amino acids (Gibco), 10 *ng/ml* LIF (ESGRO-LIF, Gibco), 0.1 *mM* β-mercaptoethanol (Sigma), 100 *U/ml* penicillin, and 50 *µg/ml* streptomycin (Gibco). ESCM was prepared when cultured embryonic stem cells reached 70% to 80% confluency. The supernatant cell culture was collected from the growing embryonic stem cells, which was defined as ESCM. This supernatant was filtered through a 0.22 *μm* membrane. A total of 15 *ml* of ESCM was placed into the tubes and centrifuged at 3000 *RPM* for 40–60 *min* and immediately used.

### Animals

A total of 20 male and 120 female NMRI mice were housed and bred in the Central Animal House of Kurdistan University of Medical Sciences. Animals were maintained on a 12 *hr* light/12 *hr* dark schedule, a temperature range of 22–24*°C*, and adequate food and water ad libitum.

### Collection and in vitro maturation (IVM) of immature germinal vesicle (GV) oocytes

**Figure 1.**
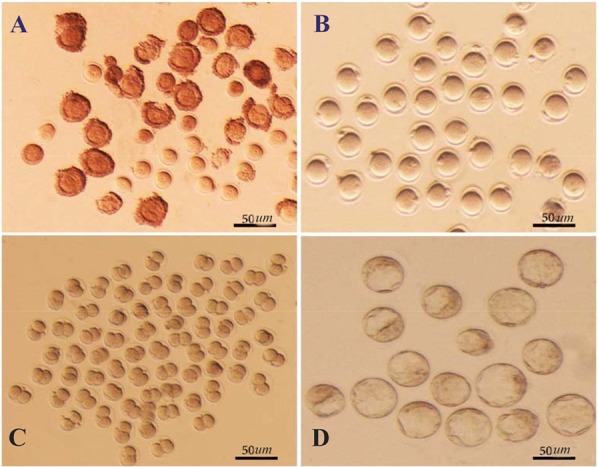
A) Immature germinal vesicle (GV) oocytes isolated from 4–6 week-old mice 48 *hr* after injection with 5 *IU* pregnant mare serum gonadotropin (PMSG), enclosed with or without compact cumulus cells (Scale bar: 50 *μm*). B) GV oocytes at 24 *hr* of culture with distinct first polar body (Scale bar: 50 *μm*). C) Developed 2-cell and D) Blastocyst embryos obtained from fertilized GV oocytes cultured in potassium simplex optimization medium (KSOM) (Scale bar: 50 *μm*).

Each 4–6 week old female mouse received an injection of 5 *IU* pregnant mare serum gonadotropin (PMSG). At 48 *hr* after the injection, immature GV oocytes from the ovaries of these mice were obtained. The GV oocytes were released from the ovaries by puncturing the follicles with a sterile 28-gauge needle as visualized under a stereomicroscope. The preantral follicles were pooled and randomly divided for further analysis (Figure 1A).

After washing human tubal fluid medium, 1199 GV oocytes were subjected to IVM. These oocytes were randomly divided, with or without cumulus cells, into three groups. The groups were placed in various maturation media and incubated at 37*°C* in a humidified atmosphere of 5% CO_2_ in air for 24 *hr*. GV oocytes were subjected to IVM in either ESCM (n=603), Embryonic Stem cell Growth Medium (ESGM, n=332), or α-Minimum Essential Medium (α-MEM, n=264). The experiments were repeated for a total of seven times per group. Oocyte maturation was evaluated at 24 *hr* by a stereomicroscope. Only those oocytes that displayed distinct first polar bodies were classified as MII oocytes. The MII oocytes underwent fertilization and *in vitro* development (Figure 1A).

### In vitro fertilization (IVF) and embryo development

The developmental potential of oocytes that had undergone Germinal Vesicle Breakdown (GVBD) or reached the MII stage *via* IVM were assessed by IVF. Sperm were collected from the cauda epididymis of mature NMRI males, capacitated for 1 to 1.5 *hr* at 37*°C*, and diluted in HTF to a final concentration of 0.7–1.3×10^6^ *sperm/ml*. MII oocytes were incubated with spermatozoa for 4–6 *hr*. Subsequently, the oocytes were washed to eliminate extra spermatozoa and then cultured overnight in separate dishes, in a drop of potassium Simplex Optimization Medium (KSOM). This medium is a conventional and chemically defined medium for development of mouse embryos. After insemination, the obtained 2pn embryos were cultured in KSOM under mineral oil at 37*°C* in an atmosphere of 5% CO_2_ and air for four days until the blastocyst stage (Figure 1D). Their developmental stages were determined by morphological evaluations conducted every 24 *hr* under a stereomicroscope. Fertilization rate was scored as the percentage of 2-cell embryos observed 24 *hr* after insemination. The maturation rate, fertilization rate, and embryo development were also determined.

### Embryo transfer

Pseudopregnant recipient mice were obtained by mating fertile female NMRI mice to vasectomized males. The following morning after mating, vaginal plugs were examined and those mice with vaginal plugs were used as pseudopregnant recipients for embryo transfer. On day 3 of pseudopregnancy (2.5 days postcoitum), the selected recipient mice were anesthetized with an intraperitoneal (*i.p.*) injection of ketamine (80 *mg/kg* body weight) and xylazine (20 *mg/kg* body weight). A 1 *cm* long skin incision was generated parallel to the dorsal midline in an attempt to expose the oviduct and uterus. An average of 10 embryos were transferred into each oviduct of the recipient mice as described by Nagy *et al* ^30^. On day 19.5 of pregnancy, recipients which did not have natural delivery were euthanized by cervical dislocation. After dissection of the uteri, the numbers of live pups and resorbed fetuses were recorded. Pups that appeared pink, and could move and breathe were identified as viable. Fully formed fetuses that did not breathe or move were considered nonviable.

### Statistical analysis

Maturation rate, IVF success, and developmental competence to the blastocyst stage in mouse oocytes were calculated for each developmental stage category and compared between the ESGM, ESCM and α-MEM groups. The data were analyzed according to the three sample test for equality of proportions with continuity correction. Birth rates for embryos matured in the study groups were analyzed by the two sample proportion test using the chi-square test with R V.3.1.0 software. P≤0.05 was considered significant.

## Results

### Effects of ESCM, ESGM, and α-MEM on the maturation of immature mouse oocytes

Each 6–8 week-old mouse received an injection of PMSG. After 48 *hr*, the oocytes were separated from the ovaries and cultured in either α-MEM, ESGM, or ESCM. Oocytes were assessed in terms of nuclear development after 24 *hr*. Nuclear maturation, indicative of resumption of meiosis from GV to the MII stage, was considered to be oocyte maturation. This stage was characterized morphologically by GVBD and the release of a distinct first polar body (Figure 1B).

No significant difference existed in maturation rates between α-MEM (68.18%) and ESCM (64.67%). However, significantly higher maturation rates were observed in α-MEM and ESCM compared to ESGM (32.22%; p=0.000). Table 1 shows the percentage of mature follicles which contained mature oocytes.

**Table 1.** Maturation rate, *in vitro* fertilization (IVF) success, and developmental competence at the blastocyst stage in mouse oocytes

**Group**	**GV oocytes (N)**	**PB N (%)**	**IVF success N (%)**	**Blastocyst N (%)**
**α-MEM**	264	180 (68.18)	125 (69.44)	64 (51.2)
**ESCM**	603	390 (64.67)	240 (61.53)	84 (35)
**ESGM**	332	107 (32.22)	0 (0.0)	-
**p-value**	-	0.000	0.000	0.0039

GV: Germinal vesicle oocyte; PB: First polar body; Metaphase II (MII); α-MEM: α-minimum essential medium; ESCM: Embryonic stem cell conditioned medium; ESGM: Embryonic stem cell growth medium.

Mature follicles were selected for *in vitro* cultivation. At 24 *hr* after IVF, the IVF success rate was determined according to the number of embryos that reached the 2-cell stage. As seen in table 1, no significant difference in IVF success rate existed between α-MEM (69.44%) and ESCM (61.53%; p=0.000). However, there was a highly significant difference for ESGM follicles (0%) compared to the success rate in IVF. There were no 2-cell stage oocytes cultured in ESGM.

### Embryo development and birth rate

In order to examine developmental ability, IVF-obtained 2pn embryos were grown to the blastocyst stage. Table 1 shows the results for *in vitro* development of mouse embryos in the different culture systems. A significantly higher percentage of blastocysts derived from α-MEM (51.2%) was observed in comparison to those derived from ESCM (35%; p=0.0039).

Embryonic viability rates at 17.5 days after embryo transfer to pesudopregnant recipient mice were evaluated (Table 1). As seen in table 2, no significant difference existed between successful birth rates for α-MEM (47.05%) and ESCM (40%; p=0.8703).

**Table 2.** Birth rates for matured embryos according to the study groups

**Groups**	**Blastocysts N**	**Birth rate N (%)**
**α-MEM**	17	8 (47.05)
**ESCM**	30	12 (40.00)
**ESGM**	-	-
**p-value**		p=0.8703

α-MEM: α-minimum essential medium; ESCM: Embryonic stem cell conditioned medium; ESGM: Embryonic stem cell growth medium.

## Discussion

This study evaluated the fate of IVM MII oocytes subjected to three different culture mediums with subsequent embryo growth. Oocyte survival in α-MEM, ESGM, and ESCM were evaluated. Our results showed that 48 *hr* after *in vivo* PMSG priming, isolated GV oocytes from 4–6 week-old mice cultured in ESCM reached a similar meiotic competence compared to those cultured in α-MEM (64 *vs*. 68%) and a higher developmental competence compared to ESGM. Furthermore, 61% of oocytes that matured in ESCM were fertilized in HTF medium from which 35% grew and reached the blastocyst stage in KSOM medium. Blastocyst transfer into pesudopregnant mice resulted in healthy pups.

According to a number of reports, embryonic stem cells secrete a variety of cytokines and growth factors which can affect different cell types ^25,29^. LaFramboise *et al* have reported that mouse embryonic stems secrete numerous proteins that mediate cardiac repair via paracrine mechanisms and cell division ^26^. Giuffrida *et al* observed that human embryonic stem cells secreted factors which arrested the growth of human epithelial, prostate, and breast cancer cell lines ^23^. According to Guo *et al*, murine embryonic stem cells released factors that stimulated the growth of normal bone marrow myeloid progenitor cells and increased survival of these cells ^25^.

Embryonic stem cells release EGF, SCF, LIF, FGF2, TGF-β, and IGF ^25,29^, which are known to support IVM of oocytes. The beneficial effects of these factors on IVM have been demonstrated in several species ^31–38^.

IGF-I stimulates oocyte maturation ^32,34–36^. According to several studies, EGF is beneficial to IVM. Oocyte maturation ^32,33^ and cumulus cell proliferation ^39^ is stimulated by EGF. The effects of EGF are possibly attributed to mitogen activated protein kinase (MAP) activities and increased H1 kinase in oocytes, mediated by cumulus cells ^40^. The beneficial effects of EGF on IVM have been demonstrated in different species, including mice ^31,33^ and humans ^32,41^.

Oocyte maturation is also stimulated by TGF-β in rats ^34^. It has been reported that maturation of both cumulus-oocyte complexes and follicle-enclosed oocytes is accelerated by TGF-β, with an increased percentage of oocytes that exhibit GVBD ^34^. Matos *et al* have stated that cumulus expansion in human and mouse cumulus-oocyte complexes were similarly induced by LIF ^42^. Maturation of sheep oocytes improved the following supplementation of the medium with FGF2 ^43^. Klinger and De Felici reported that SCF promoted oocyte growth ^44^. Therefore, an elevated IVM rate with the use of ESCM might be due to the combined effects of growth factors secreted by undifferentiated ES cells.

Similar to our results, Gelber *et al* observed that ESGM supported *in vitro* development of 8-cell embryos to blastocysts; however, when embryos were cultured from the 2-cell stage, such benefits were not observed ^45^.

Recovery of immature oocytes followed by IVM and fertilization is a potential treatment for infertility. Recently, it has been shown that IVM is a practicable alternative to conventional IVF ^46,47^.

## Conclusion

In conclusion, for the first time, the current study data showed that ESCM contained putative growth factors that could efficiently stimulate IVM of mouse oocytes, which resulted in the delivery of healthy pups following embryo transfer into recipient female mice.
